# High glucose concentrations mask cellular phenotypes in a stem cell model of tuberous sclerosis complex

**DOI:** 10.1016/j.yebeh.2019.106581

**Published:** 2019-12

**Authors:** Paula Rocktäschel, Arjune Sen, M. Zameel Cader

**Affiliations:** a Oxford Epilepsy Research Group, NIHR Oxford Biomedical Research Centre, Nuffield Department of Clinical Neuroscience, Level 6, West Wing, John Radcliffe Hospital, Oxford OX3 9DU, United Kingdom of Great Britain and Northern Ireland; b MRC Weatherall Institute of Molecular Medicine, University of Oxford, John Radcliffe Hospital, Oxford OX3 9DS, United Kingdom of Great Britain and Northern Ireland

**Keywords:** Stem cell models, Tuberous sclerosis complex, Model reliability

## Abstract

Tuberous sclerosis complex (TSC) is a neurodevelopmental disorder caused by deletions in the *TSC1* or *TSC2* genes that is associated with epilepsy in up to 90% of patients. Seizures are suggested to start in benign brain tumors, cortical tubers, or in the perituberal tissue making these tubers an interesting target for further research into mechanisms underlying epileptogenesis in TSC. Animal models of TSC insufficiently capture the neurodevelopmental biology of cortical tubers, and hence, human stem cell-based *in vitro* models of TSC are being increasingly explored in attempts to recapitulate tuber development and epileptogenesis in TSC. However, *in vitro* culture conditions for stem cell-derived neurons do not necessarily mimic physiological conditions. For example, very high glucose concentrations of up to 25 mM are common in culture media formulations. As TSC is potentially caused by a disruption of the mechanistic target of rapamycin (mTOR) pathway, a main integrator of metabolic information and intracellular signaling, we aimed to examine the impact of different glucose concentrations in the culture media on cellular phenotypes implicated in tuber characteristics. Here, we present preliminary data from a pilot study exploring cortical neuronal differentiation on human embryonic stem cells (hES) harboring a *TSC2* knockout mutation (TSC2 −/−) and an isogenic control line (TSC2 +/+). We show that the commonly used high glucose media profoundly mask cellular phenotypes in TSC2 −/− cultures during neuronal differentiation. These phenotypes only become apparent when differentiating TSC2 +/+ and TSC2 −/− cultures in more physiologically relevant conditions of 5 mM glucose suggesting that the careful consideration of culture conditions is vital to ensuring biological relevance and translatability of stem cell models for neurological disorders such as TSC.

**This article is part of the Special Issue “Proceedings of the 7th London-Innsbruck Colloquium on Status Epilepticus and Acute Seizures".**

## Highlights

• In TSC, animal models only insufficiently capture neurodevelopment and epileptogenesis.
• Human stem cell-derived cortical cultures can model cortical development in TSC.
• Culture conditions such as high glucose can mask cellular phenotypes *in vitro*.
• Careful consideration of culture conditions is vital to ensure biological relevance of stem cell models.

## 1 Introduction

Tuberous sclerosis complex (TSC) is a neurodevelopmental disorder, caused by mutations in the *TSC1* or *TSC2* genes, that is characterized by tumors in multiple organs [1]. Brain tumors, such as benign cortical tubers, as well as cortical dysorganization often lead to devastating neurological symptoms including autism spectrum disorder, learning disabilities, and seizures [2]. Epilepsy is present in up to 90% of TSC cases [3]. Seizures often start in infancy [4] with multiple seizure types reported and drug resistance in nearly two-thirds of cases [5]. The recent advent of human stem cell-based *in vitro* models has fueled hope for advances in target discovery and drug developments in TSC. However, stem cell models to study neurological disorders are still in their infancy, thereby necessitating careful consideration of the model characteristics and translational validity. Although stem cell-derived models are now used to study a variety of different brain disorders including TSC [[6], [7], [8]], the pitfalls and key characteristics of these models are still to be fully discovered and described. Certain drawbacks, such as a considerable technical variability [9] and functional immaturity of derived neurons [10,11], are already well documented. Furthermore, reliable neuronal differentiation is very dependent on cell culture media, which may support *in vitro* culture but not necessarily mimic human physiological conditions.

Studying epileptogenesis and acute seizures has generally been limited to animal tissue, mostly rodents, through the use of either *in vivo* models or *in vitro* preparations. However, research into mechanistic insights of seizure generation can be limited when using rodent models owing to significant differences in neuronal organization and brain development between rodents and humans [12]. Moreover, genetic epilepsy syndromes such as TSC are challenging to study in animal models, since pathogenic mechanisms likely originate from events during early neural development, a phase that differs profoundly between rodents and humans in terms of cell type diversity, proliferation zones, and timescales [13,14]. This translational barrier might be an important reason why mechanisms underlying human epileptogenesis are still not fully understood [15] and may, at least partly, explain why a preventative or disease-modifying antiepileptogenic therapy is not available in clinical practice, despite promising preclinical results [16]. The scientific field is, therefore, increasingly exploring the use of human-based models to better understand molecular, cellular, and developmental principles of epileptogenesis and acute seizure generation.

Stem cells entered research laboratories in the early 1980s with the exploitation of first mouse and, later, human embryonic stem cells (hES) for scientific purposes [17,18]. Since 2006, breakthrough discoveries made by Yamanaka and colleagues enabled the derivation of induced pluripotent stem cells (iPSCs) from adult somatic cells [19] and further differentiation into, theoretically, any human cell type. Thus, neuroscientists now have access to human brain cells from people with epilepsy without being dependent on specimens from brain surgery or autopsies, meaning that human-based *in vitro* models for acute seizures, epileptogenesis, and chronic epilepsy are potentially accessible. In addition, the advent of precise genome editing tools such as the CRISPR/Cas9 system [20] has made it possible to create human stem cell lines with a specific mutation of interest as well as genetically matched control lines for validating hypotheses relating to causation.

Cortical tubers and the perituberal cortex in TSC have long been implicated in the generation of seizures [[21], [22], [23], [24], [25]]. Cortical tubers are characterized by laminar dysorganization, dysmorphic neurons, eosinophilic giant cells, and severe gliosis [26]. Neither the developmental causes of this cellular dysmorphology nor the functional consequences that potentially underlie the generation of seizure activity are fully understood. Nevertheless, hyperactivation of the mechanistic target of rapamycin (mTOR) pathway is hypothesized to be a major contributor to clinical features in TSC as knockout of the TSC1:TSC2 complex deregulates the mTOR complex 1 (mTORC1) [27], and the mTOR inhibitor everolimus was recently licensed for the treatment of refractory focal epilepsy in the UK [28] and the US.

Neural differentiation protocols follow the timescale of human brain development *in vivo* so that a stem cell-based model of TSC has the potential to be particularly suited to the study of the neurodevelopmental causes for cellular malfunctioning and glioneuronal imbalance that ultimately might cause seizures in patients with TSC. Here, we present preliminary data to demonstrate that the common culture condition of a very high glucose level in culture media confounds phenotypic findings in a stem cell-based study to model TSC. Our work highlights the importance of fully defined cell culture conditions when studying disease-relevant processes and also suggests an important role for nutrient supply in TSC disease mechanisms.

## 2 Material & methods

### 2.1 Human embryonic and neural stem cell lines

We obtained hES harboring a *TSC2* deletion (TSC2 −/−) and an isogenic control line (TSC2 +/+) from La Hoffmann-Roche Limited, which were created by inserting a neomycin selection cassette into either both *TSC2* alleles or into the AAVS1 safe harbor locus of the well-characterized hES line SA001, respectively [6]. Human embryonic stem cells were kept on Matrigel-coated (Corning) cell culture plates (Corning) in mTESR (STEMCell Technologies) with daily media changes and regular passaging with Ethylenediamine tetraacetic acid (EDTA) (Invitrogen). We also obtained neural stem cells (NSCs) generated by La Hoffmann-Roche Limited from the TSC2 +/+ and TSC2 −/− hES described above [6], which were cultured in NSC media on cell culture dishes coated with poly-l-ornithine (Sigma-Aldrich) and laminin (Sigma-Aldrich).

*NSC media (25 mM “high” glucose)*: 1:1 mixture of Dulbecco's Modified Eagle Medium (DMEM)/F12 GlutaMAX (Thermo Fisher): Neurobasal medium (Thermo Fisher) supplemented with 2% B27 without vitamin A (life technologies), 1% N2 (life technologies), 50 μM β-mercaptoethanol (life technologies), 10 ng/ml brain-derived neurotrophic factor (bFGF) (Peprotech), 10 ng/ml epidermal growth factor (EGF) (R&D), and 20 ng/ml brain-derived neurotrophic factor (BDNF) (Peprotech).

*5 mM “low” glucose NSC media*: DMEM w/o glucose (Thermo Fisher) and Neurobasal-A-medium w/o glucose (Thermo Fisher) were used supplemented as above with the addition of 5 mM glucose (life technologies).

No ethical approvals were required for this research, which utilised established stem cell lines. Approval for use of the established embryonic stem cell lines was granted by the Steering Committee of the UK Stem Cell Bank.

### 2.2 Differentiation of TSC2 +/+ and TSC2 −/− hES

Neuronal differentiation was carried out as first described by Shi et al. [29]. At 100% confluency, hES were induced with neural induction media (NIM) and kept in NIM with daily media changes for 10–12 days. Afterwards, the neuroepithelial sheet was manually passaged in clumps onto cell culture plates coated with 20 μg/ml laminin. Between day 12 and 18, cultures were kept in neural maintenance media (NMM) supplemented with 20 ng/ml bFGF (Peprotech) with media changes every other day. At around day 18, neural rosettes were manually passaged in NMM on 20 μg/ml laminin. Until day 35, full NMM changes were performed every other day, and cells were passaged with accutase (STEMCell Technologies) between day 22 and 25 if they reached 80–100% confluency. At around day 35, a final plating was performed with accutase in NMM on 20 μg/ml laminin at a plating density of 30,000 cells/cm^2^. From then onwards, cells were kept until day 80–100 with full NMM changes every other day.

*Neural maintenance media (NMM, 25 mM “high” glucose)*: 1:1 mixture of DMEM/F12 GlutaMAX:Neurobasal medium supplemented with 1% B27 (Invitrogen), 0.5% N2, 50 μM β-mercaptoethanol, 1 mM l-glutamine (Thermo Fisher), 25 U/ml penicillin–streptomycin (Thermo Fisher), 2.5 μg/ml insulin (Sigma-Aldrich), 0.5 μM sodium pyruvate (Sigma-Aldrich), and 50 μM MEM nonessential amino acids (life technologies).

*5 mM “low” glucose NMM*: DMEM w/o glucose and Neurobasal-A-medium w/o glucose were used supplemented as above with the addition of 5 mM glucose.

*Neural induction media (NIM)*: NMM supplemented with 1 μM dorsomorphin (Tocris) and 10 μM SB431542 (Tocris).

### 2.3 Growth curves

Growth curves were obtained for TSC2 +/+and TSC2 −/− NSCs in their high or low glucose culture conditions. Wells were marked with a cross at the bottom, and a picture from each quadrant in the middle of the plate was taken every day between 24 h after plating and reaching 100% confluency. Images were analyzed with ImageJ/FIJI. Doubling time (Dt) was calculated as

$$\mathrm{Dt}=\frac{\log2∙\mathrm{duration}}{\log\left(\mathrm{final cell count}\right)-\log\left(\mathrm{initial cell count}\right)}.$$

### 2.4 Western blotting

In brief, cells were gathered in Radioimmunoprecipitation assay buffer (RIPA) (Thermo Scientific) supplemented with phosphatase–proteinase inhibitor (Thermo Scientific) and kept at 4 °C overnight. Eight micrograms of sample were run on NuPAGE 4–12% Bis-Tris gels (Invitrogen) in MOPS SDS Running Buffer (life technologies) and transferred to a nitrocellulose membrane using the Bio-Rad Trans-Blot Turbo Transfer System. Blots were blocked in 5% bovine albumin serum (BSA, Sigma-Aldrich) in TBS-T buffer (Bio-Rad) and subsequently incubated in primary antibodies in 5% BSA in Tris-buffered Saline (Bio-Rad) with 0.1% Triton X-100 (Sigma-Aldrich) (TBS-T buffer) at 4 °C overnight. The next day, blots were incubated in secondary antibodies in 5% BSA. Enhanced luminol-based chemiluminescent (ECL) detection was performed using the Pierce™ ECL Western Blotting Substrate (Thermo Scientific). Images were processed and analyzed using ImageJ/FIJI. The following primary antibodies were used: anti-TSC2 (rabbit, Cell Signaling, 1:500), anti-pS6 (rabbit, 1:2000, Cell Signaling), anti-S6 (rabbit, Cell Signaling, 1:8000), horseradish peroxidase (HRP)-conjugated anti-Actin (mouse, Abcam ab49900, 1:25,000), and HRP-conjugated anti-rabbit (goat, Abcam ab6721, 1:8000).

### 2.5 Immunofluorescence

Cultures were fixed on coverslips with 4% paraformaldehyde (PFA, Alfa Aesar) and permeabilized with 0.1% Triton X-100 (Sigma-Aldrich) in Phosphate-buffered saline (PBS). After blocking in 10% normal goat serum, cells were incubated with primary antibody in 5% normal goat serum overnight at 4 °C. The following day, cultures were incubated with secondary antibodies in 5% normal goat serum and stained with 600 nM 4',6-Diamidino-2-Phenylindole (DAPI). Images were processed with ImageJ/FIJI. The following antibodies were used: anti-Glial fibrillary acidic protein (GFAP) (chicken, 1:2000, Abcam ab4674), anti-TUJ1 (rabbit, 1:2000, Abcam ab18207), anti-HuC/HuD (mouse, 1:1000, life technologies), AF488-conjugated anti-chicken (goat, 1:1000, Abcam ab150169), AF488-conjugated anti-mouse (goat, 1:1000, Abcam ab150113), and AF594-conjugated anti-rabbit (goat, 1:1000, Abcam ab150080).

### 2.6 Flow cytometry for cellular composition

Cells were gathered at different timepoints during neuronal differentiation with accutase, and the cell pellet was fixed in 4% PFA and permeabilized in 100% methanol. For staining, methanol-stored samples were incubated with primary antibodies in fluorescence-activated cell sorting (FACS) buffer for 1 h (1% fetal bovine serum (FBS) 10 μg/ml Immunoglobulin G (IgG) from human serum in PBS) and after washes in FACS buffer subsequently incubated in the respective secondary antibodies in FACS buffer for 20 min and analyzed on a CYAN ADP. The following antibodies were used: AF647-conjugated anti-GFAP (mouse, BioLegend 644706, 1:400), AF488-conjugated anti-Tubulin b (mouse, BioLegend 801203, 1:200), BV421-conjugated anti-Nestin (mouse, BioLegend 656808, 1:100), anti-HuC/HuD (mouse, life technologies, 1:400), AF488-conjugated anti-Ki67 (mouse, EMD Millipore MAB4190, 1:400), and AF488-conjugated anti-mouse (goat, 1:1000, Abcam ab150113).

## 3 Results

### 3.1 Neuronal differentiation protocols

**Fig. 1:**
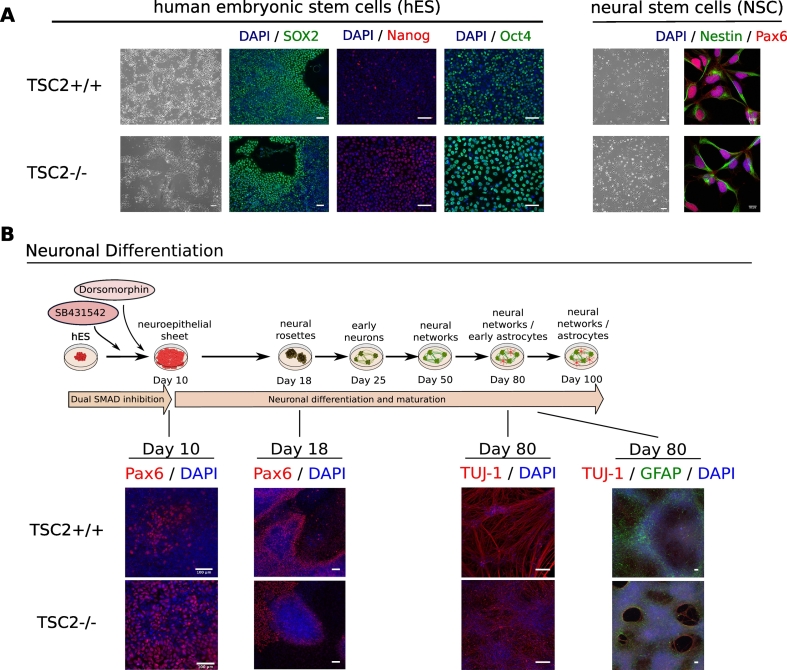
Characterization of TSC2 +/+ and TSC2 −/− cell lines. (A) A hES line harboring a *TSC2* knockout (TSC2 −/−) and an isogenic control line (TSC2 +/+) were generated at La Roche-Hoffmann Ltd. and NSCs derived from these hES lines were characterized immunologically. No differences could be observed in the expression of the pluripotency markers SOX2, Nanog, and Oct4 in the TSC2 +/+ and TSC2 −/− hES. Also, TSC2 +/+ and TSC2 −/− NSCs expressed the NSC markers Nestin and Pax6 in a similar manner. (B) Neuronal differentiation of TSC2 +/+ and TSC2 −/− hES was performed by dual SMAD inhibition for 80 days. After 10 days, the neuroepithelial sheet consisting of Pax6-positive NSCs had formed in both cultures followed by Pax6-positive neural rosettes around day 18. Rosettes in TSC2 −/− cultures tended to be bigger and cell-denser as in wildtype cultures. After 80 days of maturation, both lines generated TUJ1-positive neurons and few GFAP-positive astrocytes. However, TSC2 −/− neuronal cultures were found to be denser despite a similar initial plating density.

Immunofluorescent characterization of TSC2 +/+ and TSC2 −/− hES and NSC lines showed no obvious morphological differences between the wildtype and the knockout cell lines with regard to pluripotency at the hES stage and NSC markers (Fig. 1A). During neuronal differentiation, both cell lines produced Pax6-positive neuroepithelial cells around day 10 and Pax6-positive radial glial cells at the neural rosette stage around day 18. After 80 days, cortical neurons were generated in both cultures. However, TSC2 −/− showed a higher proliferation potential producing denser and bigger rosettes as well as denser neuronal populations (Fig. 1B).

### 3.2 Necessity to use low glucose media to exacerbates mTOR phenotype

**Fig. 2:**
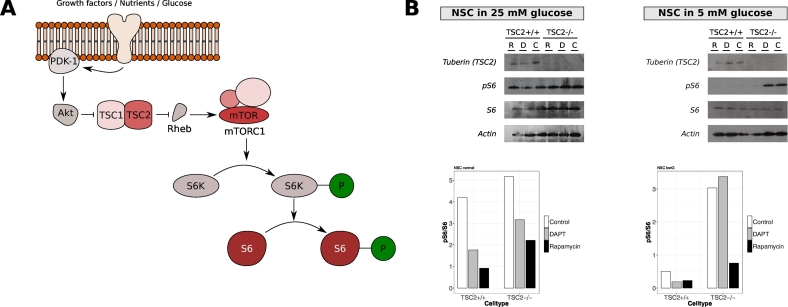
Effect of different glucose levels on mTORC1 activation in NSCs. TSC2 +/+ and TSC2 −/− NSCs cells in their standard media with either 25 mM or 5 mM glucose were examined for protein levels of tuberin (TSC2), S6, phosphorylated S6 (pS6), and actin as loading control. (A) As activation of mTORC1 increases phosphorylated S6 (pS6), the activity level of the mTORC1 can be determined by measuring the ratio of phosphorylated S6 to total S6 (pS6/S6). (B) Protein extracts were obtained after 24 h of treatment with either 5 nM rapamycin (R), 10 μM DAPT (D), or DMSO (C) as vehicle control in either 25 mM or 5 mM glucose media (n = 1 for each condition). In 25 mM glucose media, no differences in derived mTORC1 activation could be observed in TSC2 +/+ and TSC2 −/− NSCs as the pS6/S6 ratios were not altered. This is despite the absence of tuberin in TSC2 −/− cells. The level of mTORC1 activation was reduced by rapamycin. In contrast, 5 mM glucose media nearly abolished mTORC1 activity (no pS6 detected) in TSC2 +/+ NSCs. In TSC2 −/− NSCs, S6 was phosphorylated although this phosphorylation could be attenuated by rapamycin.

Activation of mTORC1 can be measured by the ratio of phosphorylated S6 (pS6) to total S6 (Fig. 2A). Protein levels for tuberin (gene product of TSC2), pS6, and S6 were detected by western blotting in TSC2 +/+ and TSC2 −/− NSCs in either 25 mM or 5 mM glucose media (Fig. 2B). No differences were observed in the pS6/S6 ratio between wildtype and knockout NSCs in their conventional media. However, after 24 h in culture media containing 5 mM glucose, TSC2 +/+ NSCs significantly lowered their mTORC1 activation, whereas TSC2 −/− NSCs maintained a similar mTOCR1 activity level as in the 25 mM glucose media. As expected, mTORC1 could be inhibited by treatment with 5 nM rapamycin for 24 h in all cultures and media.

### 3.3 Glucose levels affect proliferation and cellular features in TSC2 cultures

**Fig. 3:**
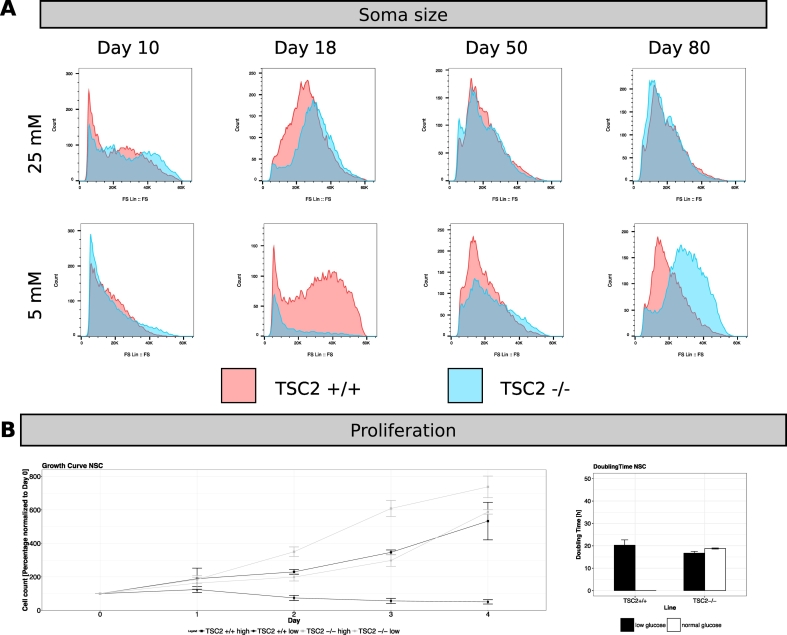
Effect of different glucose levels on soma size and proliferation during neuronal differentiation. (A) Flow cytometry signals of the forward scatter (FS Lin) as a measure of cell size for TSC2 +/+ (red) and TSC2 −/− (blue) cultures during Livesey differentiation at timepoints days 10, 18, 50, and 80 in high and low glucose conditions (n = 1 for each differentiation). No differences in cell size between the genetic conditions could be seen in 25 mM glucose media. However, soma size differed in 5 mM glucose media at the NSC (day 18) and neuronal (day 80) stage with TSC2 −/− cells being smaller at day 18 but larger at day 80 than TSC2 +/+ cells. (B) Growth curves obtained from TSC2 +/+ and TSC2 −/− NSCs. Cells were cultured for 3–4 days in 25 mM culture media or 5 mM culture media (n = 9 for each condition). In high glucose media, TSC2 −/− NSCs doubled in a similar time to TSC2 +/+ NSCs (TSC2 +/+: 18.8 h, TSC2 −/−: 16.7 h). No differences in growth curve and doubling time could be observed in TSC2 −/− NSCs when transferred to 5 mM glucose media (20.3 h), whereas a reduction in glucose concentration completely abolished proliferation of TSC2 +/+ NSCs. (For interpretation of the references to color in this figure legend, the reader is referred to the web version of this article.)

We next examined whether cellular phenotypes such as soma size and proliferation rates were also impacted by glucose concentration. Soma size was examined at different timepoints during neuronal cortical differentiation, using media containing either 25 mM or 5 mM glucose, by flow cytometry analysis of the forward scatter (FS) profile (Fig. 3A). TSC2 +/+ and TSC2 −/− cells showed no differences in cell size during neuronal differentiation in high glucose media. However, reducing the glucose concentration to 5 mM altered cell size at day 18 in both cell lines and at day 80 in TSC2 −/− cultures.

We then investigated the impact of glucose on cellular growth of TSC2 +/+ and TSC2 −/− NSCs in either 25 mM or 5 mM glucose media. In low glucose media, TSC2 +/+ NSCs stopped proliferating completely after an initial drop in cell number. In contrast, the growth of TSC2 −/− NSCs was not affected by the lower glucose concentration with no change of the Dt after changing to low glucose media (Fig. 3B).

### 3.4 TSC2 knockout affects cellular composition in low glucose media

To detect how *TSC2* knockout impacts cell fate choices during neuronal differentiation in the context of different glucose concentrations, we performed cortical neuronal differentiations on TSC2 +/+ and TSC2 −/− hES in either 25 mM or 5 mM glucose media and examined the expression of the neuronal marker TUJ1; the glial maker GFAP; the NSC marker Nestin, and the postmitotic neuronal marker HuC/HuD at day 10, day 18, day 50, and day 80. We also characterized cell cycle activity throughout the differentiation by measuring the levels of Ki67 that marks cells actively progressing through the cell cycle.

**Fig. 4:**
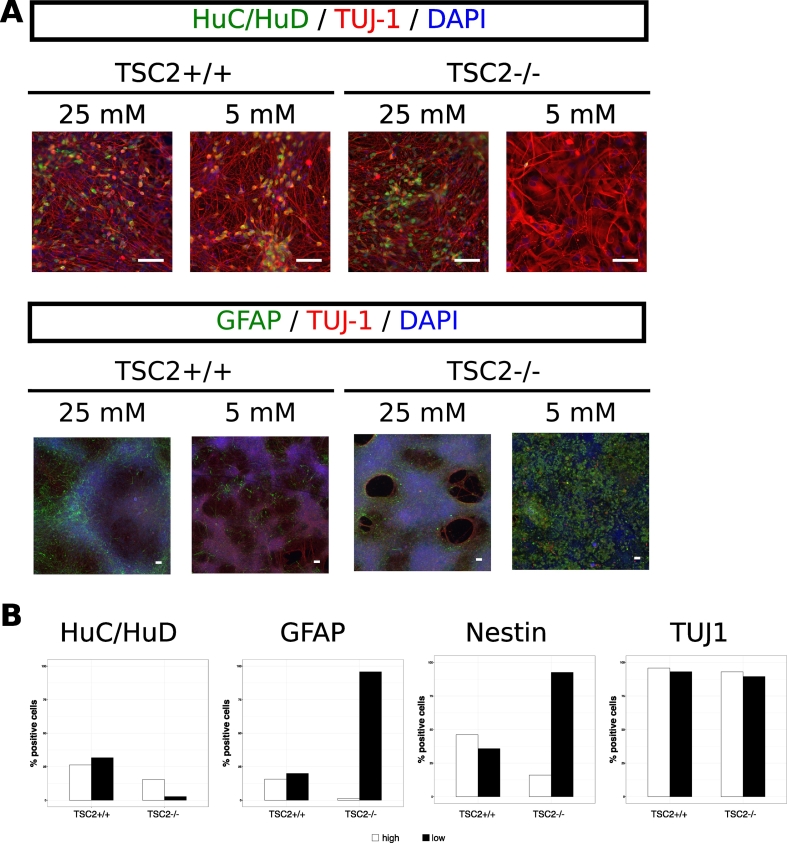
Impact of a low glucose concentration on cellular output in neuronal differentiation. Cortical neuronal differentiation was performed on TSC2 +/+ and TSC2 −/− hES in either 25 mM or 5 mM glucose media. Cellular compositions were examined at day 80 using immunofluorescence (A) and flow cytometry (B) for the postmitotic marker HuC/HuD; neuronal marker TUJ-1 and glial marker GFAP (n = 1 for each differentiation). For HuC/HuD, no differences were observed in TSC2 +/+ cultures between the differentiations in high or low glucose as both media led to the generation of approximately 25% postmitotic neurons at day 80 and TSC2 −/− cells produced just under 20% HuC/HuD-positive neurons in 25 mM glucose media. However, lowering the glucose concentration to 5 mM nearly abolished the generation of postmitotic neurons in TSC2 −/− cultures. The opposite could be observed with regard to GFAP-positivity, which did not differ for TSC2 +/+ cultures in both media, whereas GFAP-positivity increased to nearly 95% in TSC2 −/− cultures kept at 5 mM glucose. No differences between cell lines and media were found for TUJ1, which was detected in approx. 95% of all cells in all cultures.

**Fig. 5:**
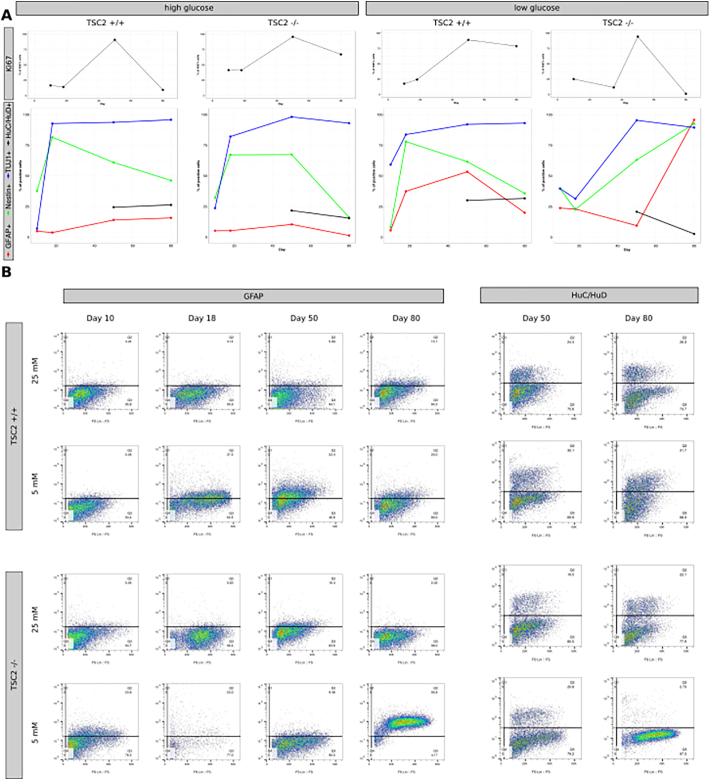
Characterization of neuronal differentiation of TSC2 +/+ and TSC2 −/− cells in 25 mM and 5 mM glucose media. (A) Positivity for the proliferation marker Ki67 and the cell fate markers GFAP, Nestin, TUJ1, and HuC/HuD was measured using flow cytometry during cortical neuronal differentiation at days 10, 18 (Ki67 at day 35 in TSC2 −/− cultures in 5 mM glucose), 50, and 80. In 25 mM glucose media, TSC2 +/+ and TSC2 −/− cells expressed differentiation markers similarly. Cell cycle activity increased between the neural progenitor stage at day 18 and day 50 after which most TSC2 +/+ cells entered a cell cycle inactive state. Ongoing proliferation beyond day 50 could be observed in TSC2 −/− cultures. Lowering the glucose level changed the expression profile of the differentiation markers in TSC2 −/− cultures, whereas the differentiation of TSC2 +/+ cells was not affected. TSC2 −/− cells in low glucose media lacked the decline in Nestin expression and were characterized by an increase in GFAP expression from day 50 onwards. The proportion of HuC/HuD-positive cells declined from 19.5% at day 50 to 2.7% at day 80. In contrast to the 25 mM glucose condition, cell cycle activity could be detected in 75% of TSC2 +/+ cells at day 80, whereas TSC2 −/− completely lost their cell cycle activity after day 50 (n = 1 for each differentiation). (B) Example flow cytometry plots for GFAP and HuC/HuD for TSC2 +/+ and TSC2 −/− cultures in 25 mM and 5 mM glucose media at respective timepoints showing no significant difference between cell lines throughout the whole differentiation regardless of media, except for TSC2 −/− cells in 5 mM glucose media at day 80 with an increase in the GFAP-positive and a decrease in the HuC/HuD-positive population.

TSC2 +/+ showed an expected profile for these differentiation markers during neuronal differentiation with a rapid increase in TUJ1 and a decline in Nestin after the neural progenitor state as well as no significant expression of GFAP. Furthermore, 24.4% and 26.2% of the culture were positive for HuC/HuD at day 50 and day 80, respectively. Cell cycle activity peaked after formation of the neural rosettes at the neural progenitor stage and declined as expected at the differentiation stage after day 50. The change in glucose concentration did not alter these marker profiles in TSC2 +/+ cultures with the only exception from this being an ongoing cell cycle activity at day 80 in low glucose media (Fig. 5A). In contrast, glucose concentration strongly impacted on cell fate choices in TSC2 −/− cultures that followed the wildtype marker profile in 25 mM glucose media but showed severely disturbed expression of differentiation markers in 5 mM glucose media. The expression of HuC/HuD was particularly affected and was completely abolished under low glucose conditions at day 80, alongside a marked increase in GFAP expression to 95.8% at the last timepoint (Fig. 4). Moreover, TSC2 −/− cells lost cell cycle activity at day 80 in the low glucose media.

## 4 Discussion

In this report, we show preliminary data from a stem cell-based approach to the study of TSC highlighting the importance of using culture conditions that are as relevant as possible to the *in vivo* human physiology. We compared cellular proliferation and cell fate choices during cortical neuronal differentiation of TSC2 +/+ and TSC2 −/− hES in high and low glucose media. Our data indicate that commonly used high glucose concentrations *in vitro* might mask important cellular phenotypes in a stem cell model of TSC.

In high glucose conditions, we found no phenotypic differences between TSC2 +/+ and TSC2 −/− NSCs with regard to cell size and differentiation potential. Only the proliferation rate was increased during differentiation in *TSC2* knockout cultures. Higher proliferation rates have been reported in TSC2 −/− NSCs previously [6] although other studies could not confirm this observation [30]. However, lowering the glucose concentration to more physiological levels increases the differences in neuronal differentiation potential between TSC2 +/+ and TSC2 −/− cultures — more closely resembling the glioneuronal imbalance and disturbed neuronal development that can be found in cortical tubers *in vivo*.

In patients with TSC, acute seizure activity likely originates from cortical tubers and the perituberal cortex [[21], [22], [23]], which are characterized by a glioneuronal imbalance, the presence of dysmorphic neurons, and eosinophilic giant cells [26]. Hyperactivation of the mTOR pathway has been implicated in the development of cortical tubers as mTORC1 has a well-established role in cell proliferation and cell fate choices [31]. However, molecular mechanisms of tuber formation are difficult to study in animal models as, so far, no animal model of TSC develops an equivalent to cortical tubers [13]. Early knockout of either *TSC1* or *TSC2* in rodents is lethal necessitating a conditional knockout of *TSC1* or *TSC2* in either neurons or astrocytes at later stages of neurodevelopment in animal models [32]. Furthermore, fundamental differences in cortical development between humans and rodents might also contribute to the absence of cortical tubers in rodent models. Hence, human-based models are likely necessary to study molecular and cellular mechanisms relevant to tuber development and epileptogenesis in patients with TSC.

The differentiation and maintenance of stem cell-based neuronal cultures are now widely adopted in the study of various neurological diseases. However, a thorough characterization of culture conditions and their impact on cell fate choices and cellular function is not well explored — to some extent because media compositions are not often declared and there may be a reluctance to alter systems that appear to enable adequate differentiation. In our model system, we culture and differentiate stem cell-derived neurons in fully defined media based on frequently used basal media such as DMEM and Neurobasal media. The basal media contain nonphysiological high glucose concentrations to keep the cultured cells well-supplied with nutrients. However, we have now shown that the high concentration of glucose can mask relevant phenotypes such as cell fate choices, proliferation, and cell size. These phenotypes become apparent when culturing and neuronally differentiating TSC2 −/− hES with a glucose concentration of 5 mM that more closely resembles the glucose levels in the human body.

Further investigations are now warranted to confirm our findings and better understand the mechanisms for disturbed cell fate choices in TSC2 −/− cultures. We might speculate that TSC2 −/− cells have lost their ability to match cell metabolism to nutritional supply by failing to downregulate the mTORC1 pathway. This might then impact on the ability to activate correct signaling pathways in neuronal differentiation. When using conventional media compositions, high glucose in the media may mask this mismatch as TSC2 −/− cells would still have a sufficient supply of glucose for their increased metabolism.

As in every model system, it is vital to design the most appropriate model parameters to balance high-quality reproducible results and biological relevance. More broadly, our work highlights the importance of considering and fully reporting protocols and media system ingredients to ensure meaningful and reproducible results [33]. As other limitations of stem cell-based studies (such as immaturity of derived neurons [10], high variability [9], difficulties in obtaining appropriate iPSC control lines, and the challenge to replicate neurodevelopment in 3-dimensional structures such as brain organoids [34]) are increasingly addressed, close attention to culture media composition will increasingly support the reliability of such models and improve their future clinical relevance.
